# Pro- and antisaccades in children elicited by visual and acoustic targets - does modality matter?

**DOI:** 10.1186/1471-2431-11-116

**Published:** 2011-12-16

**Authors:** Johanna Goepel, Stefanie C Biehl, Johanna Kissler, Isabella Paul-Jordanov

**Affiliations:** 1 Department of Clinical Psychology, University of Konstanz, 78457 Konstanz, Germany; 2 Department of Psychiatry, Psychosomatics, and Psychotherapy, University of Würzburg, 97080 Würzburg, Germany; 3 Department of Psychologiy, Universtity of Bielefeld, 33501 Bielefeld, Germany

## Abstract

**Background:**

Children are able to inhibit a prepotent reaction to suddenly arising visual stimuli, although this skill is not yet as pronounced as it is in adulthood. However, up to now the inhibition mechanism to acoustic stimuli has been scarcely investigated

**Methods:**

Reflexive (prosaccade) and inhibitory (antisaccade) responses to visual *and *acoustic targets were examined with an eye tracker system in 31 children between seven and twelve years of age using a gap-overlap task and two target eccentricities.

**Results:**

Acoustically cued saccades had longer reaction times than visually cued saccades. A gap effect (i.e., shorter reaction time in the gap than the overlap condition) was only found for visually elicited saccades, whereas an eccentricity effect (i.e., faster saccades to more laterally presented targets - 12° vs. 6° or rather 90° vs. 45°) was only present in the acoustic condition. Longer reaction times of antisaccades compared to prosaccades were found only in the visual task. Across both tasks the typical pattern of elevated error rates in the antisaccade condition was found. Antisaccade errors declined with age, indicating an ongoing development of inhibitory functions.

**Conclusions:**

The present results lay the ground for further studies of acoustically triggered saccades in typically as well as atypically developing children and it might thus be possible to upgrade physiological diagnostic tools.

## Background

It is a reflex-like feature of human behaviour to look towards sudden changes in our visual field. This enables us to respond adequately to changes in our environment. Scientifically, this reflexive behaviour is studied with prosaccade tasks. Here, participants are required to generate a saccade to a suddenly appearing peripheral visual target - also called "visual grasp reflex". Parameters such as accuracy and saccadic reaction time (SRT) can be measured [1]. In order to not look towards a suddenly appearing peripheral target, volitional inhibition of the visual grasp reflex is required. Scientifically, this can be investigated with antisaccade tasks [2]. Here, participants are asked to suppress a prosaccade towards a visual target and to look at its mirror position in the opposite visual field instead. As antisaccades require active inhibition of an already initiated motor response, more direction errors are made and SRTs are longer compared to prosaccade tasks [3-8]. The timing between the central target offset versus the peripheral target onset affects direction error rate and SRT of both pro- and antisaccades. When the central fixation cross disappears before the onset of the peripheral target (gap condition), more errors are produced than when the peripheral target appears while central fixation is still visible (overlap condition). At the same time, SRT in gap conditions is reduced compared to overlap conditions. This "gap effect" is probably due to the reduction in firing rate of fixation neurons in the superior colliculus and frontal eye fields with gap onset [7-9], which is called the "disengagement of ocular fixation hypothesis". Next to this hypothesis, an advanced movement preparation in the gap task relative to the overlap task is discussed as the underlying physiological mechanism [10]. This causes faster responses as saccade neurons in these structures start firing earlier. The SRT gap effect is bigger for prosaccades than for antisaccades [4] and more pronounced in children than in young adults [3,7,11]. Another factor affecting direction errors and SRT is the peripheral position (eccentricity) of the target. Both the number of direction errors in response to visual targets and SRT increase with larger stimulus eccentricity [12,13]. Studies of ocular motor performance in children have shown that SRT decreases with age [14] as does the proportion of direction errors, although at a different pace [3,6,7,11,15].

Humans do not only look towards visual stimuli, they also direct their gaze to locate the origin of a suddenly appearing sound. This reaction is already present in babies [16]. Although saccades towards acoustic targets are scientifically less well investigated than saccades towards visual targets, a recent study delineated an "acoustic-evoked ocular grasp reflex" in adults [17].

Both children and adults also need to be able to inhibit reflexive visual responses to acoustic stimuli. A child, for example, will automatically look at the person who calls his name. But standing in the middle of a busy street it might be better to not look at the person but to focus on the traffic coming from the opposite direction to avoid an accident. An important difference between the visual and auditory modality is the reference system. While input to the visual field is thought to be processed in relation to a retinotopic reference system, acoustic targets are related to a craniotopic, i.e., head-related, reference system [13]. The craniotopic reference system is by definition wider than the retinotopic reference system, this being caused by the position of the ears on the sides of our head, while the eyes face forward. The craniotopic reference system is more accurate and sensitive to lateral stimuli, while the retinotopic system is most accurate for stimuli directly in front of us. When sounds trigger a saccadic response, it is assumed that sound representation needs to be remapped from the craniotopic into the retinotopic reference system, in order to produce spatially correct saccades [12].

Animal studies with nonhuman primates as well as experimental studies with adults have revealed lower accuracy and longer SRTs of prosaccades towards acoustic targets than towards visual targets [18-20]. This is probably caused by the additional demand of remapping from the craniotopic to the retinotopic reference system. Considering target eccentricity, a reverse relationship between SRTs and target position has been found in the auditory compared to the visual modality: SRTs of acoustically triggered saccades decrease for larger stimulus eccentricities, but SRTs of visually triggered saccades increase with larger stimulus eccentricities [12,13,21,22]. Ostensibly, at least in adults, a processing advantage for centrally presented visual stimuli and a disadvantage for centrally presented acoustic stimuli exists [19]. In adults, the gap effect also interacts with target modality. The gap effect regarding SRTs of prosaccades appears less pronounced for acoustic than for visual targets [22-25].

The vast majority of studies on saccades triggered by acoustic targets only investigated prosaccades. Until now there are only two studies, which investigated acoustically triggered antisaccades in adults [17,26]. One of these studies [17] studied acoustic antisaccades in three patients with hemispherectomy and a control group. They revealed that patients generated more errors and showed longer SRTs than control participants. Schooler and colleagues [26] investigated adults with and without schizophrenia and compared performance in antisaccade tasks using visual and acoustic targets. They found a higher error rate for acoustically than visually triggered antisaccades in healthy young adults while patients generated the reverse pattern of more errors during visually than during acoustically elicited antisaccades.

The present study is - to the best of our knowledge - among the first studies comparing SRTs and error rates of pro- and antisaccades elicited by visual and acoustic targets in typically developing children. We investigated children between seven and twelve years of age regarding the impact of central fixation engagement (gap, overlap) as well as target eccentricity on pro- and antisaccades elicited by visual and acoustic peripheral targets.

This is of relevance as studies on pro- and antisaccades triggered by acoustic and visual targets in children will further our understanding of modality differences in ocular motor responses and the development of basic, ecologically as well as clinically relevant sensory-motor processing assessments.

## Methods

### Ethics Statement

Ethical approval was obtained through the Ethical Review Board of the University of Konstanz. All procedures involved were in accordance with the 2008 Declaration of Helsinki [27]. Children and parents gave written informed consent after full explanation of the procedures.

### Participants

31 children between seven and twelve years of age participated in this study. They were recruited at primary schools in the Konstanz area. Six children had to be excluded because they were too small for the eye-tracker, too restless, had a partial hearing loss, low scores on a questionnaire on auditory processing and perception, or because they decided to not to continue after the half-time break in the experiment. The 25 remaining children (18 girls and seven boys) had a mean age of 9.31 ± 0.24 years. 24 children were right-handed, one child was left-handed. None of the children fulfilled criteria for attention deficit hyperactivity (ADHD) or auditory processing disorder (APD) and their parents did not report any other neurological, psychiatric, or physiologic problems.

### Procedure

The families were shown the laboratory equipment and the task was explained to them. Parents were asked to fill in a general information questionnaire about their child, an ADHD symptom checklist [28], and an auditory processing disorder checklist [29] while children completed the Edinburgh-Handedness-Inventory [30]. To ensure within-normal hearing levels, children's hearing thresholds were determined for frequencies 500, 1000, 2000 and 4000 Hz in an acoustically shielded room. Children were then shown a computerised, animated explanation of the task, which included examples and four training trials. For additional motivation, children were told that they would be able to collect four "cartoon dogs" on the computer screen if they performed well (although the dogs always appeared after fixed intervals) which would then allow the children to pick a small gift from a "treasure chest" after the experiment. Thus, it was ensured that all children were motivated and perceived themselves as successful. Children were additionally compensated with 10 Euros at the end of the experimental session.

For the eye-tracker experiment, children were comfortably seated on a height-adjustable chair, their heads resting on a chin rest 518 mm away from the computer monitor. Brightness and contrast of the eye tracker-camera were adapted, headphones were put on and the 30 min - experiment was started after calibration of the eye tracker (11 standard positions distributed over the screen).

### Task

Participants were instructed to generate saccades in response to visual or acoustic targets presented in two randomised blocks with 144 trials each. The nature of the required saccade depended on the instruction symbol. Saccades could either be directed towards the target (prosaccade) or away from the target (antisaccade). Visual targets consisting of yellow dots that filled one of four empty circles could appear "less eccentric" (6°) or "more eccentric" (12°) to the left or right of the fixation cross for 1000 ms. Acoustic targets were 1000 Hz sine tones presented for 1000 ms that were perceived either "more eccentric" left/right (90°) or "less eccentric" left/right (45°, see the description below). Children were instructed that in response to "less eccentric" acoustic targets they should generate saccades towards the 6° circle, and upon "more eccentric" to make saccades towards the 12° circle. Targets could either appear 200 ms after extinction of the fixation cross (gap) or with a 200 ms overlap with the fixation cross. Random trial combinations of the following within-group factors were presented throughout the experiment: target modality (visual vs. acoustic), direction (right vs. left), type (anti- vs. prosaccade), distance (less eccentric (6° visual, 45° acoustic) vs. more eccentric (12° visual, 90° acoustic)), delay (gap vs. overlap). Nine runs of each combination resulted in a total of 288 trials.

After trials 96, 129, 259 and 288 children were shown a motivation picture with 1, 2, 3 and 4 dogs, respectively. A pause-signal appeared after 144 trials indicating that children could take a short break. The length of the break was determined by the children.

**Figure 1:**
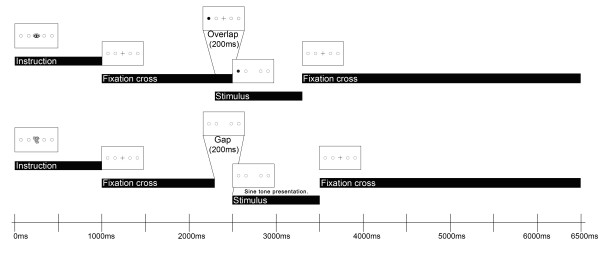
**Example trial (prosaccade)**. top: visual overlap-condition and bottom: acoustic gap-condition.

Each trial began with a 1000 ms instruction slide depicting the nature of the required saccade by a prominent symbol (an ear for acoustically cued prosaccades, an eye for visually cued prosaccades and crossed symbols for antisaccades) the meaning of which had been explained to the children beforehand (see procedure above). Each trial lasted 6500 ms (see Figure 1 for a schematic overview).

### Equipment and Oculomotor Recordings

Targets were presented with the software Presentation (Neurobehavioral Systems, Inc.). Visual targets were generated within Presentation. Sine tones were generated with Adobe Audition 2.0^®^. The effect of sound lateralisation was created by intensity and phase differences between the left and right channel. The impression of a 90° lateralisation to either direction was created by attenuating the contra-lateral channel by 3.62 dB and shifting its onset by 6.5 μs. The impression of a 45° lateralisation was created by attenuating the contralateral channel by 2.8 dB and delaying its onset by 1 μs.

Stimuli were presented with a PC (Intel (R) Pentium (R) 4, CPU 3.00 GHz processor, 522.928 RAM) running a Windows 2000^® ^operating system on a monitor with 640 × 480 pixels resolution (22"/51 cm viewable; Iivama MA203DT; Vision Master Pro 513) and via stereo headphones (Sony Digital Reference Dynamic MDR-CD470). The recording computer had the same specifications as the stimulus computer.

Eye movements were measured with a high-speed camera system (iView Hi-Speed-Eye Tracker, SensoMotoric Instruments, Teltow, Germany). The eye-tracker had a temporal resolution of 240 Hz and a spatial resolution < 0.01°. Data were stored for offline analysis. During testing, eye movements were visualised on the recording computer to enable on-line monitoring and re-calibration, if necessary.

### Data analysis

SRTs and direction of saccades were analysed. Saccade onset was defined semi-automatically with the software BeGaze^® ^Version 1.02.0076 (SensoMotoric Instruments, http://www.smivision.com). Individual saccades were cross-checked manually and onsets were corrected if necessary.

There were very few trials (a mean of 20.6 trials per child) where no reaction could be detected. Since the small amount of these trials did not warrant a separate analysis. Secondary and anticipatory saccades were excluded from further analysis.

Direction error rate and SRT were analysed statistically using Statistica version 6.1 (StatSoft, Inc., 2003, http://www.statsoft.de). Univariate repeated measures analyses of variance (ANOVAs), using within-subject factors modality (visual/acoustic), type (anti-/prosaccade), distance (less/more eccentric), and delay (gap/overlap) were computed. Significant interactions were investigated further with a post hoc test (Tukey's Honest Significant Difference-Test). Correlations between age (in months) and dependent variables were tested using the Bravais Pearson correlation test and Spearman Rank test. Correlations of dependent variables in the visual and acoustic condition were tested with the Bravais Pearson correlation test, Spearman Rank test and the partial correlation test.

## Results

### Saccadic Reaction Times

Results will be restricted to correct trials, since incorrect trials were rare in some conditions (i.e., visual prosaccades), potentially skewing latency results. There were no significant correlations between age and SRTs in either condition.

**Figure 2:**
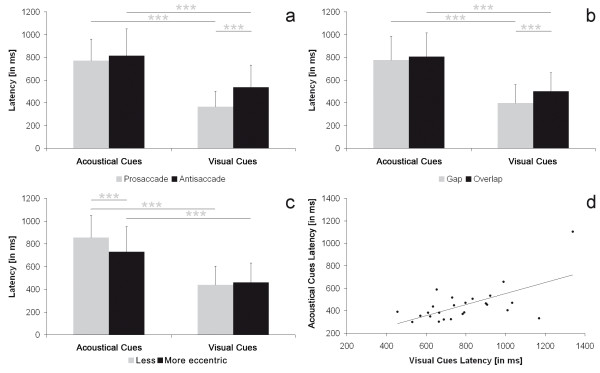
**Results of latency**. a: Interaction modality*type for the dependent variable latency; b: Interaction modality*delay for the dependent variable latency; c: Interaction modality*distance for the dependent variable latency; d: Correlation between latencies in visually and acoustically cued saccades; filled circles: antisaccades, empty circles: prosaccades.

Therefore, it was not necessary to use age as a covariate of no interest in the ANOVAs. Mean SRT for correct reactions was 621 ± 163 ms. Saccades triggered by visual targets had shorter SRTs than saccades after acoustic targets (main effect modality F(1, 23) = 97.36, p < .001, acoustic: 791 ± 202 ms, visual: 449 ± 160 ms); SRTs of prosaccades were shorter than SRTs of antisaccades (main effect type F(1, 23) = 20.49 p < .001, pro: 567 ± 144 ms, anti: 675 ± 196 ms). An interaction between modality and type was found (F(1, 23) = 18.61, p < .001, see Figure 2a). Acoustically triggered pro- and antisaccades did not differ in SRTs (acoustic-pro: 769 ± 191 ms, acoustic-anti: 813 ± 236 ms, p = .20). In contrast, SRTs of visual prosaccades were significantly shorter than SRTs of visual antisaccades (visual-pro: 365 ± 136 ms, visual-anti: 535 ± 196 ms, p < .001).

SRTs in gap conditions were shorter than in overlap conditions (main effect delay: F(1, 23) = 29.99, p < .001, gap: 588 ± 166 ms, overlap: 654 ± 165 ms). The interaction modality*delay (F(1, 23) = 16.40, p < .001, see Figure 2b) showed no SRT difference between gap and overlap for saccades triggered by acoustic stimuli (acoustic-gap: 776 ± 208 ms, acoustic-overlap: 806 ± 209 ms, p = .61), whereas SRTs in gap-conditions were shorter than in overlap conditions for visually evoked saccades (visual-gap: 397 ± 162 ms, visual-overlap: 501 ± 163 ms, p < .001, Figure 2b).

Investigating SRT as a function of stimulus eccentricity showed that SRTs after targets less eccentric to the fixation cross were longer than SRTs after targets that were further away (main effect distance F(1, 23) = 7.75, p < .05, less eccentric: 645 ± 162 ms, more eccentric: 596 ± 174 ms). A significant interaction modality*distance was also found (F(1, 23) = 19.21, p < .001, see Figure 2c). Less and more eccentric stimulus targets led to equally long SRTs within visually triggered conditions (visual-less eccentric: 438 ± 167 ms, visual-more eccentric: 461 ± 171 ms, p = .68), whereas for acoustically evoked saccades SRTs were shorter after targets that were further away from the fixation cross compared to less eccentric targets (acoustic-less eccentric: 853 ± 198 ms, acoustic-more eccentric: 729 ± 223 ms, p < .001).

There was a positive correlation between SRTs in the visual and the acoustic condition (r(1, 23) = .52, p < .01) across saccade type and for pro- and antisaccades, respectively (antisaccades: r(1, 23) = .50, p < .05; prosaccades: r = .47, p < .05, see Figure 2d).

### Error Rates

As there was a correlation between age and overall-error rate (age/error r = -.46, p < .05), as well as age and antisaccade errors (see table 1), age was used as continuous predictor in the ANOVAs.

**Table 1:** Correlation of errors with age [in month]

Age [in month] correlation with		r(X.Y)	p
All		-0.46	0.01

Antisaccades	All	-0.48	0.00
	Visual	-0.47	0.02
	Acoustic	-0.36	0.07

Prosaccades	All	-0.14	0.46
	Visual	-0.01	0.97
	Acoustic	-0.24	0.26

**Figure 3:**
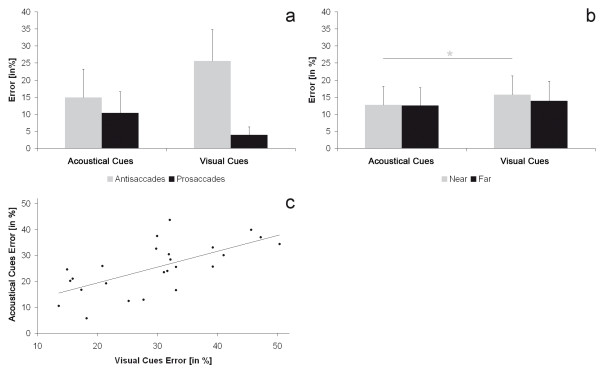
**Results of error rate**. a: Interaction modality*type for the dependent variable error rate; b: Interaction modality*distance for the dependent variable error rate; c: Correlation between errors in visually and acoustically cued saccades.

Across all conditions a mean of 27.38 ± 9.22% direction errors were generated, whereof 69.05 ± 12.31% were corrected. The main effect type revealed that children made more direction errors during anti- than prosaccades (antisaccades: 20.27 ± 7.42%, prosaccades: 7.11 ± 3.61%, F(1, 23) = 12.43, p < .01). The interaction type*modality was not significant (F(1, 23) = 3.38, p = .08; visually cued antisaccades: 25.58 ± 9.29%, acoustically cued antisaccades 14.88 ± 8.34%, visually cued prosaccades: 3.93 ± 2.35%, acoustically cued prosaccades 10.35 ± 6.25%, see Figure 3a for the error rate per modality condition). The trend shows higher error rates in the anti- than in the prosaccades in the visual condition but not in the acoustic condition and additionally higher error rates during saccades towards acoustic than visual targets within the prosaccade condition and an opposite result within the antisaccade condition.

The interaction modality*distance (F(1, 23) = 4.62, p < .05, see Figure 3b) revealed that modalities only differed in the 8° condition (p < .05). Error rates were higher during visually than during acoustically elicited saccades (visual condition: 15.67 ± 5.58%, acoustic condition: 12.74 ± 5.38%). No further post-hoc tests were significant.

There was a positive correlation between errors in the visual and the acoustic condition (r(1, 23) = .65, p < .001, see Figure 3c) across saccade type, and this was still significant in a partial correlation corrected for age (r(1, 23) = .58, p < .01). However, further inspection revealed that error rates only correlated between modalities for antisaccades without controlling for age, not for prosaccades (antisaccades: r(1, 23) = .40, p < .05, partial correlation: r(1, 23) = .30, p = .13; prosaccades: r(1, 23) = .27, p = .16).

## Discussion

The present study is among the first studies investigating pro- and antisaccades following visual and acoustic targets in normally developing children.

### Visual targets

Using visual targets, the present study replicates a number of previous findings: SRTs were longer for antisaccades than for prosaccades [4,5,7,15,31] and shorter for gap than for overlap trials [7,15,31]. More direction errors were made on antisaccade than on prosaccade trials [5-8]. Target eccentricity affected neither RT nor error rate. This corresponds with findings in a study with children that used similar eccentricities as the present one, namely 8°, 12° and 24° [6], but differs from other studies with adults [12,13], suggesting that developmental effects may account for the null finding in children. Alternatively, the missing effects of target eccentricity might be explained by overlaps of the saccade amplitude because of the small visual angles. The analysis of amplitude gain might have yielded information about the overlap of the saccade amplitudes depending on the eccentricity but this was not analysed within the scope of this work.

In comparison to other studies (e.g. [32]) the latencies of visually cued saccades in this study were very long. Reason for that could be the mixed method of stimulus presentation that required a permanent interpretation of the cue and updating of the task instruction [33] - a process needing time.

### Acoustic targets

Using acoustic targets revealed that SRTs did not differ between pro- and antisaccades. An explanation might be that as the remapping process from the craniotopic to the retinotopic reference system takes more time (as reflected in over-all longer SRTs for acoustically triggered saccades), the salience of the acoustic target becomes obscured and thus the immediate "grasp-reflex" less strong. This is somewhat supported by the findings regarding error rates. Although the main effect condition suggests that across modalities more antisaccade errors were made than prosaccade errors, the interaction type*modality was not significant. Thus, although there is a tendency for more errors in the anti- than in the prosaccade condition, the anti/pro-difference is smaller than in the visual condition. This is due to more prosaccade and fewer antisaccade errors in the acoustic than the visual condition. Correct acoustic prosaccade generation appears to be more difficult than visual prosaccade generation in children. In line with this finding, a lower error rate on acoustically than on visually cued antisaccades was observed by Schooler and colleagues [26] in adult schizophrenia patients but not in healthy adults. The authors offered the explanation that the remapping process reduces immediate inhibitory demands on the system, making the acoustic stimulus "less preemptive". Therefore, people with reduced executive system capacities may experience a relative benefit from different modality targets on tasks requiring response inhibition. Children's executive system is also less developed, as frontal lobe maturity is only reached in adulthood [34]. Therefore their lower error rate in the acoustic antisaccade task might result from their presumably less developed executive system, which may benefit from the extra time gained in the re-mapping process.

No auditory gap effect was found for SRTs or error rates. In contrast to this, previous findings in adults have described a smaller, but still significant gap effect for acoustically cued saccades [17,23-25]. It might be the case for children that although mean SRTs indicate a gap-effect, it is obscured by a relatively high SRT variablity (208 ms gap, 209 ms overlap condition). Alternatively, Fendrich and colleagues [23] suggest that a reduction (or absence) of the gap effect for acoustic targets might be due to the fact that gap durations are usually chosen to be optimal for visual but not necessarily for auditory saccades. In line with this notion and the present findings, Reuter-Lorenz and colleagues [17] also did not find a gap effect for error rates when antisaccades were elicited by acoustic targets.

Target eccentricity had an effect on SRTs of acoustically cued saccades with saccades to more peripheral targets being generated more quickly than saccades to the less eccentric targets. This extends previous results in adults [12,13,19,20,22] to children between the ages of seven and twelve.

### Comparison between visual and acoustic targets

Comparing results across modalities showed that SRTs to acoustic targets were generally longer than to visual targets. This finding replicates previous results for prosaccades in adults [13,18-20,22-24] and extends them to children and to antisaccades. It shows that extra processing time is needed to switch between reference systems. Target eccentricity affected SRTs to acoustic, but not visual targets, saccades to more distant acoustic targets being generated more quickly than to closer targets. Presumably, within a craniotopic reference system more lateral targets are easier to locate than less eccentric targets [12]. Modality differences were found in the less eccentric condition: more errors for visually than acoustically triggered saccades were made. One possible explanation for this result might be the difference in the eccentricity of the presented stimulus between modalities. While visual targets were presented 8° or 12° lateral of the fixation cross, acoustic stimuli represented a 45° and 90° angle, respectively. Thus, it might have been easier to distinguish stimulus directions in the acoustic condition.

Correlations between age (in months) and SRTs revealed no developmental effects of age on saccadic RT within the age range studied. Yet, across all conditions the slope of the regression line was negative, still indicating a small reduction of SRTs with age until young adulthood, which, in line with other previous reports, may reach significance for wider age ranges [3,7,11,35]. For direction errors, significant negative correlations between age in months and error percentage were observed for both visual and acoustic antisaccades, but not for prosaccades, indicating a significant improvement in antisaccade performance between the ages of seven and twelve years in both modalities. These results extend the findings of developmental visual saccade performance [3,11] to the auditory modality. Thus, it can be assumed that developmental effects are comparable for the visual and the acoustic condition.

The correlation between latencies in visual and acoustic conditions indicates that the children's abilities in both tasks are to some degree comparable supposable. Children with slower latencies in visual condition also had slower latencies in the acoustic condition. The same was true for error rate.

## Conclusion

The present study was the first study to investigate pro- and antisaccades elicited by visual and acoustic stimuli in normal developed children. While many similarities between target presentation modalities arose, there were important differences: the "grasp-reflex" was weaker for auditory saccades and auditory saccades seemed less prone to the influence of impulsivity as their latency was longer resulting in fewer antisaccade errors. Studying different input modalities in the context of response inhibition might be of interest for investigating populations with disorders such as auditory processing disorder (APD) and attention-deficit/hyperactivity disorder (ADHD).

## Competing interests

The authors declare that they have no competing interests.

## Authors' contributions

JG carried out the subject selection, data acquisition, data processing, statistics and the preparation of the manuscript.

SB carried out the subject selection, data acquisition and data processing.

JK made substantial contribution to study design, data analysis and the manuscript.

IPJ designed the study, carried out statistics and corrected the manuscript.

All authors read and approved the final manuscript.

## Pre-publication history

The pre-publication history for this paper can be accessed here:

http://www.biomedcentral.com/1471-2431/11/116/prepub
